# *Sphaerillo boninensis* Nunomura, 1990 (Crustacea, Isopoda, Oniscidea) is a junior synonym of a pantropical species, *Venezillo parvus* (Budde-Lund, 1885)

**DOI:** 10.3897/zookeys.923.26018

**Published:** 2020-04-01

**Authors:** Shigenori Karasawa

**Affiliations:** 1 Department of Life and Environmental Agricultural Sciences, Faculty of Agriculture, Tottori University, 4-101 Koyama-machi Minami, Tottori 680-8553, Japan Tottori University Tottori Japan

**Keywords:** Armadillidae, Chichijima Island, Ogasawara archipelago, terrestrial isopods, UNESCO World Heritage Site

## Abstract

Re-examination of the holotype and paratype of *Sphaerillo boninensis* Nunomura, 1990 from Chichijima Island of the Ogasawara archipelago, which is registered as a UNESCO World Heritage Site, indicates that this species is a junior synonym of a pantropical species, *Venezillo parvus* (Budde-Lund, 1885).

## Introduction

*Sphaerillo boninensis* Nunomura, 1990, described from specimens collected on Chichijima Island, Ogasawara archipelago, is a small terrestrial isopod formerly considered endemic to this archipelago, a UNESCO World Heritage site approximately 1,000 km from the mainland of Japan. However, there are questions around the taxonomy of this species. The genus that this species has been attributed to has changed many times (see the taxonomic account; Nunomura 1990, 1999a, 2011), and the species is included in the genus *Spherillo* in the current taxonomic treatment (Nunomura 2015). In addition, Saito (1993) recorded specimens collected from Chichijima Island and identified as the pantropical species *Venezillo parvus* (Budde-Lund, 1885) by Drs Ferrara and Taiti, but this species has been overlooked in reviews of Japanese terrestrial isopod species (Nunomura 1999a, b, 2015; Saito et al. 2000).

*Venezillo parvus*, originally and imperfectly described as *Armadillo parvus* by Budde-Lund (1885), was subsequently transferred to the genus *Venezillo* Verhoeff, 1928 (Green et al. 1990; Taiti and Ferrara 1991a). Today *Venezillo* accommodates more than 140 recognized species (Boyko et al. 2008). More comprehensive, subsequent descriptions of *V. parvus* provided more useful taxonomic characteristics than those present in the original description, i.e., in Schultz (1963) as *V. evergladensis* Schultz, 1963, a species now considered to be a junior synonym of *V. parvus*, Ferrara & Taiti, 1983 as *Sphaerillo* (?) *parvus* (Budde-Lund, 1885), Schmidt (2003) and Gregory (2014) as *V. parvus*. Figures provided by Schmidt (2003) depicted the basis of the male pereopod 7 as bearing dense setae on the anterior corner of the ventral side, a characteristic present also on the male pereopod 7 of *S. boninensis* (Nunomura 1990, fig. 146I). Additionally, the oblique lobe on the ventral surface of pereonite 2 reported for *S. boninensis* by Nunomura (1990, fig. 146B) is a diagnostic feature of the genus *Venezillo* (Verhoeff 1928; Vandel 1952). These similarities indicated that *S. boninensis* was referable to *Venezillo* and a close relationship between *S. boninensis* and *V. parvus* might exist.

Terrestrial isopods are now scarce in the southern part of Hahajima Island, Ogasawara archipelago, in an area invaded by the land nemertean *Geonemertes pelaensis* Semper, 1863 (Shinobe et al. 2017). This terrestrial predator poses a threat to native biodiversity in this region. Resolving the systematic status of *S. boninensis*, or at least specimens referred to it, from this area would improve our understanding of actual versus perceived threats to endemic faunas of this region, and ultimately, the conservation and management of species throughout this archipelago.

The aim of this study was to resolve relationships between *S. boninensis* and *V. parvus*. This is achieved through re-examination and redescription of appropriate type material of the former.

## Materials and methods

### Sample collection

Type material was loaned from the Toyama Science Museum (collection acronym TOYA). As the previously dissected male holotype and paratype of *S. boninensis* were in poor condition, a description of the whole body, cephalon, antenna 1, and pleopod 5 of the female paratype was necessary. Additionally, as the male pereopods 1 and 7 are broken in male holotype, they can be only partly described. Male pereopod 2 was not found in the holotype and paratype.

### Morphology observation

Antennae 1 and 2, mandible, maxillae 1 and 2, maxilliped, pleopods, and pereopods were placed in Hoyer’s mounting medium (Krantz and Walter 2009) on slides, covered with a coverslip, and drawn under a microscope (magnification of 40–400 ×; Eclipse E400, NIKON Corp.). The whole body, cephalon, and epimera of pereonites 1–7 were examined using a Nikon SMZ1500 microscope (magnification of 7.5–112.5 ×).

## Results

### Taxonomic account

**Genus *Venezillo* Verhoeff, 1928**

#### Venezillo parvus

Taxon classificationAnimaliaIsopodaArmadillidae

(Budde-Lund, 1885)

62C6099F-71FD-54CB-A98D-31E50CCE87AE

Armadillo parvus Budde-Lund, 1885: 25–26; Dollfus 1893: 187; Verhoeff 1946: 4; Jeppesen 2000: 254.
Spherillo parvus : Budde-Lund 1904: 91; Budde-Lund 1908: 270–271, taf 12, figs 30–37; Budde-Lund 1912: 371; Barnard 1964: 53.
Venezillo evergladensis Schultz, 1963: 209–213, figs 1–26; Schultz 1972: 1–4, figs 4–6; Schultz 1975: 186; Johnson 1976: 157, fig. 1; Johnson 1977: 603; Johnson 1978: 140; Johnson 1980: 124; Johnson 1981: 351, fig. 1; Johnson 1982: 225; Johnson 1983: 209; Johnson 1984: 465; Johnson 1985a: 403; Johnson 1985b: 216; Keeney 1990: 1–2, figs 1, 2.
Sphaerillo parvus : Ferrara and Taiti 1979: 182.
Sphaerillo (?) parvus: Ferrara and Taiti 1981: 196; Ferrara and Taiti 1983: 70–71, figs 131–136; Taiti and Ferrara 1983: 220–222.
Sphaerillo boninensis Nunomura, 1990: 19–21, fig. 146. Syn. nov.
Venezillo parvus : Green et al. 1990: 431, 433; Taiti and Ferrara 1991a: 220; Taiti and Ferrara 1991b: 915; Kwon and Taiti 1993: 77; Kwon 1995: 533; Taiti and Howarth 1996: 68; Taiti et al. 1998: 300; Leistikow and Wägele 1999: 49; Taiti 1999: 38; Jass and Klausmeier 2000: 781; Stoyenoff 2001: tab. 2; Green et al. 2002: 301; Zimmer et al. 2002: 597; Buck et al. 2003: 106; Kelly and Samways 2003: app 1; Schmidt 2003: 107, figs 122–128; Soesbergen 2003: 97, figs 1, 2, tab. 1; Schmalfuss 2004: 291; Zimmer et al. 2004: 754, figs 1–3, 5–12; Dias et al. 2005: tab. 3; McLaughlin et al. 2005: 198; Poore 2005: 11; Berg et al. 2008: 61; Gregory 2009a: 7; Gregory 2009b: 1; Barber 2010: 73; Cochard et al. 2010: tab. 7.1.1; Senterre et al. 2011: app 2; Murphy et al. 2012: 3; Arab and Wimp 2013: 333; Treplin et al. 2013: 84; Wimp et al. 2013: 507, fig. 1; Angelini and Silliman 2014: 188; Gregory 2014: 21–23, fig. 11, tab. 3; Humphreys 2014: tab. 2; Lemaire and Gerriet 2014: 43–44; Taiti and Wynne 2015: 44; Taiti 2018: 88; Vittori and Štrus 2017: 396; De los Rios-Escalante et al. 2018: 50, tab. 1; Pérez-Schultheiss et al. 2018: 8.
Venezillo boninensis : Nunomura 1999a: 89; Nunomura 1999b: 598, 614, 624; Saito et al. 2000: 94. Syn. nov.
Spherillo boninensis : Nunomura 2011: 71; Nunomura 2015: 1032, 1050, 1063; Shinobe et al. 2017: 3. Syn. nov.

##### Material examined.

*Sphaerillo boninensis*: Holotype, TOYA-Cr-8953, male, dissected, forest of *Casuaria equisetifolia*, Suzaki, Chichijima Island, Tokyo Metropolis, Japan, 1 July 1977, Jun-Ichi Aoki leg; paratype, TOYA-Cr-8955, male, dissected, forest of *Casuaria equisetifolia*, Suzaki, Chichijima Island, Tokyo Metropolis, Japan, 1 July 1977, Jun-Ichi Aoki leg; paratype, TOYA-Cr-8961, female, dissected, forest of *Casuaria equisetifolia*, Suzaki, Chichijima Island, Tokyo Metropolis, Japan, 1 July 1977, Jun-Ichi Aoki leg.

### Redescription of *Sphaerillo boninensis* Nunomura, 1990

**Figure 1.**
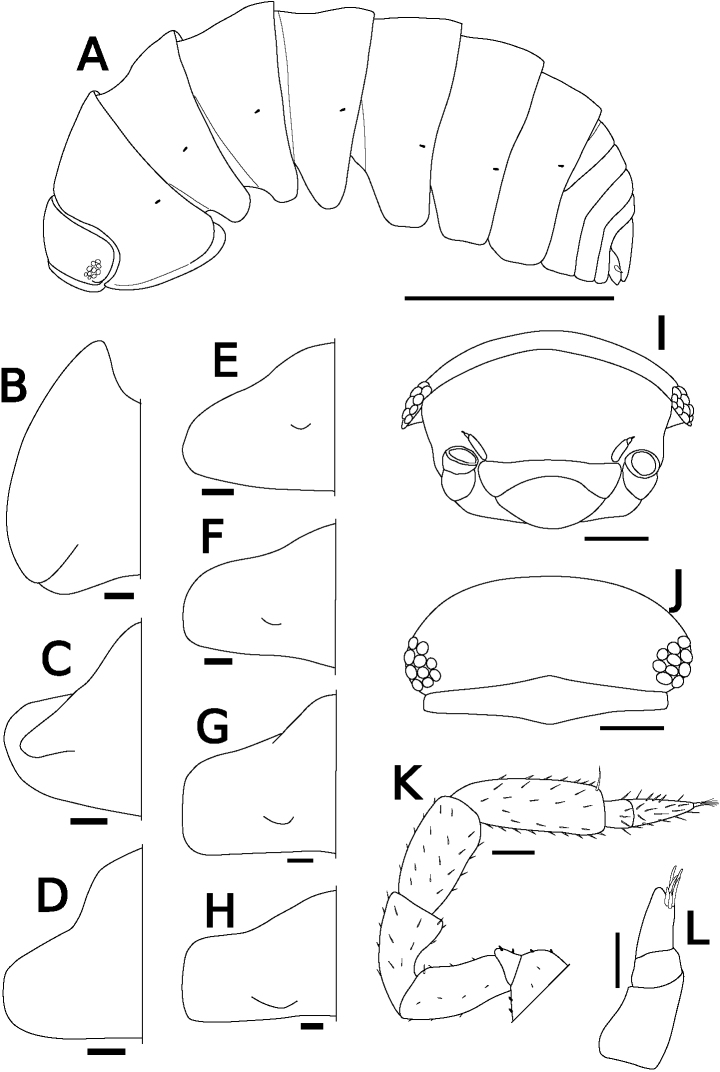
*Sphaerillo boninensis***A** whole body, lateral view, paratype (female) **B–H** epimeron of pleonites 1–7, ventral view, holotype (male) **I** cephalon, frontal view, paratype (female) **J** cephalon, dorsal view, paratype (female) **K** second antenna, holotype (male) **L** first antenna, paratype (female). Scale bars: 1.5 mm (**A**); 100 μm (**B–H, K**); 300 μm (**I, J**); 50 μm (**L**).

**Figure 2.**
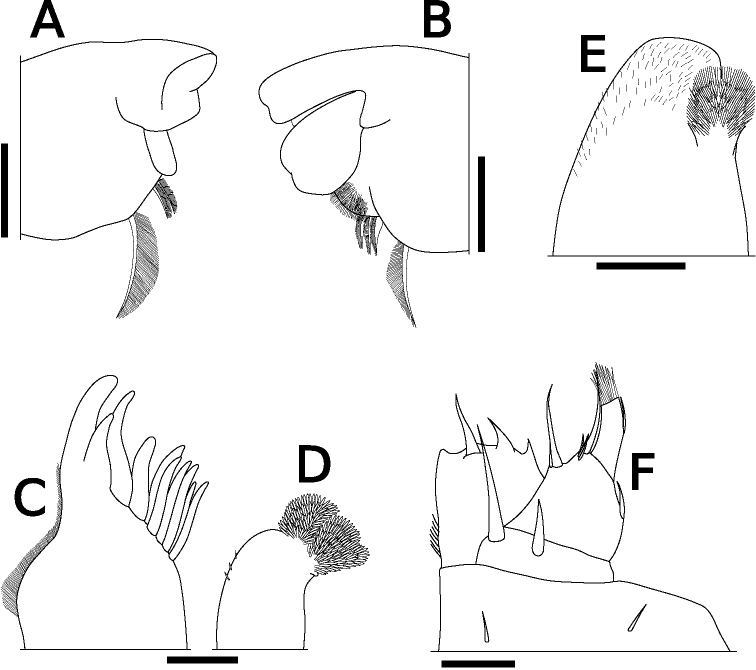
*Sphaerillo boninensis***A** right mandible, holotype (male) **B** left mandible, holotype (male) **C** outer endite of first maxilla, holotype (male) **D** inner endite of first maxilla, holotype (male) **E** second maxilla, holotype (male) **F** maxilliped, holotype (male). Scale bars: 100 μm (**A, B**); 50 μm (**C–F**).

**Figure 3.**
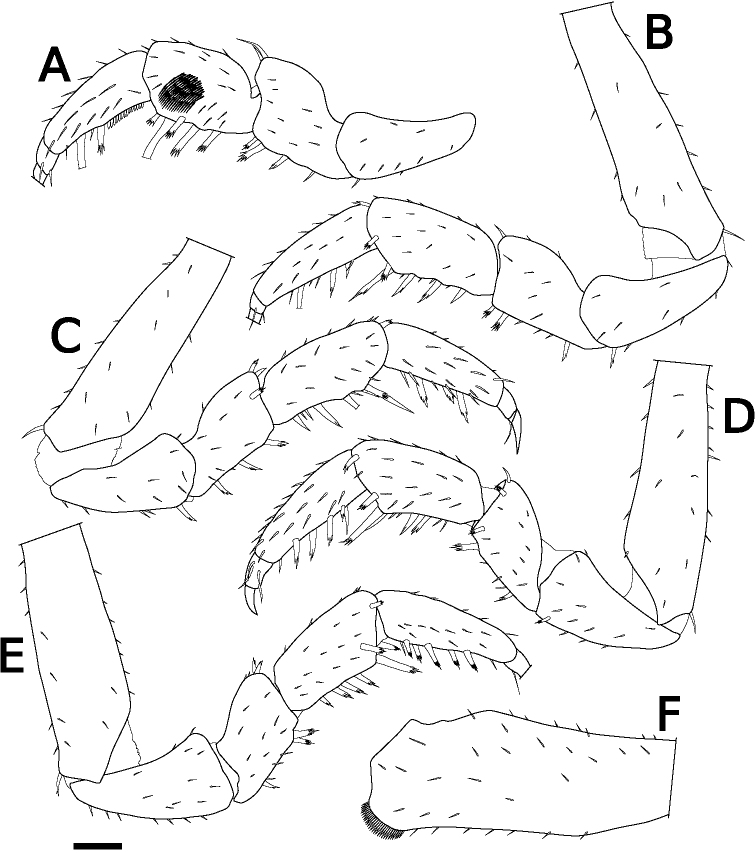
*Sphaerillo boninensis***A** pereopod 1, holotype (male) **B–E** pereopods 3–6, holotype (male) **F** basis of pereopod 7, holotype (male). Scale bar: 100 μm.

**Figure 4.**
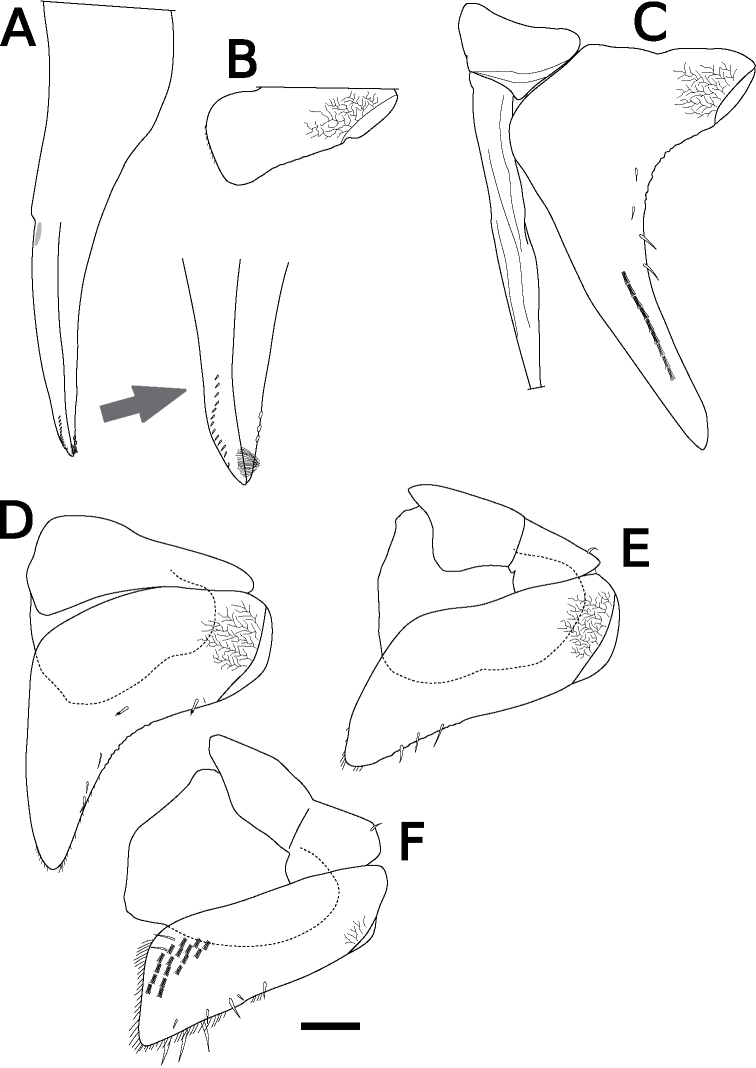
*Sphaerillo boninensis***A** endopodite of pleopod 1, holotype (male) **B** exopodite of pleopod 1, holotype (male) **C–E** pleopods 2–4, holotype (male) **F** pleopod 5, paratype (female). Scale bar: 100 μm.

**Figure 5.**
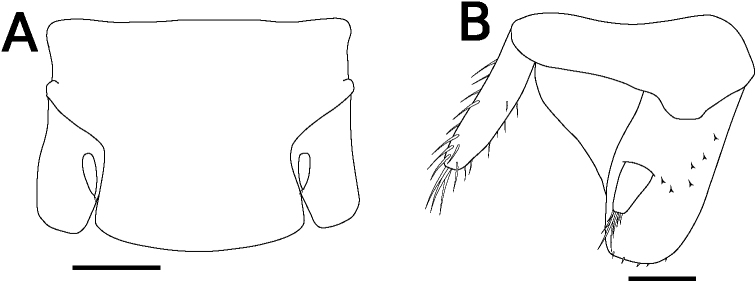
*Sphaerillo boninensis***A** pleotelson, holotype (male) **B** uropod, holotype (male). Scale bars: 200 μm (**A**); 100 μm (**B**).

Body color yellowish in alcohol. Pereonites 1–7 with single nodulus lateralis per side, all similarly distanced from lateral margin (Fig. 1A). Pereonite 1 with lateral margin not grooved; schisma deep, with rounded inner and outer lobes, outer lobe protruding posteriorly compared to inner lobe (Fig. 1A, B). Pereonite 2 with an oblique lobe on ventral surface (Fig. 1C). Pereonite 3 without ventral lobe (Fig. 1D). Pereonites 4–7 with a small lobe on ventral surface (Fig. 1D–H). Eyes (of female paratype) with ten ommatidia (Fig. 1I, J). Upper middle edge of cephalon convex; frontal shield separated from vertex, trapezoidal in frontal view (Fig. 1I, J). Flagellum of second antenna with two articles (Fig. 1K). First antenna of three articles; apical article with numerous aesthetascs (Fig. 1L). Right mandible with two plumose setae between lacinia mobilis and molar penicil; lacinia mobilis of left mandible larger than right mandible; molar penicil unbranched; left mandible with three plumose setae between hairy lobe and molar penicil (Fig. 2A, B). First maxilla outer endite with ten simple teeth; inner endite with two stout plumose penicils (Fig. 2C, D). Second maxilla apically bilobate, covered with short setae (Fig. 2E). Endite of maxilliped rectangular, bearing three spines on distal margin; maxilliped palp with basal article bearing two long setae, distal article with apical tuft of small setae (Fig. 2F). Male pereopod 1 with antennal brush on carpus; propodus with numerous short setae on basal half of inner margin; carpus with five long stout setae on inner margin; apical point of outer margin of merus with two tapering setae, inner margin with three branched setae (Fig. 3A). Male pereopods 3–6 unremarkable (Fig. 3B–E). Basis of male pereopod 7 with dense setae on apical corner of inner margin (Fig. 3F). All pleopod exopodites with monospiracular covered lungs (Fig. 4). Male pleopod 1 with straight endopodite, with apical part bent outward with long setae; inner margin with row of small setae; outer margin with at least three tooth-like setae (Fig. 4A); exopodite triangular, with small setae on inner margin (Fig. 4B). Male pleopod 2 endopodite slender (apical part broken); exopodite triangular, with distal half elongated (Fig. 4C). Male pleopod 3 exopodite with triangular part on posterior inner corner (Fig. 4D). Male pleopod 4 exopodite parallelogram-shaped (Fig. 4E). Female pleopod 5 parallelogram-shaped, with fine setae of variable length along inner margin (Fig. 4F). Pleotelson hour-glass-shaped, distal part narrower than basal part (Fig. 5A). Uropodal protopod trapezoidal, with exopodite inserted on dorsal surface of protopod; endopodite about twice as long as exopodite (Fig. 5B).

## Discussion

The genus *Venezillo* is characterized among other characters by a narrow lobe located obliquely or horizontally on the ventral surface of the pereon epimeron 2 (Verhoeff 1928; Vandel 1952). The holotype of *S. boninensis* has an oblique lobe on the ventral surface of the epimeron 2, for which reason it is more appropriately assigned to the genus *Venezillo*. In addition, in all other morphological characteristics, type materials of *S. boninensis* are consistent in morphology with those of *V. parvus* as redescribed by Schmidt (2003): the apical corner of the basis of the male pereopod 7 bears dense short setae (Fig. 3F), the endopodite of the male pleopod 1 has long setae at the tip and scale-like setae on the inner margin (Fig. 4A), and the exopodite of the male pleopod 1 is triangular (Fig. 4B). For these reasons, I regard *S. boninensis* to be a junior synonym of *V. parvus*, a species widely distributed in the tropical belt.

While *S. boninensis* was considered endemic to the Ogasawara archipelago, and potentially threatened by an invasive nemertean predator (Shinobe et al. 2017), the pantropical *V. parvus*, due to its widespread distribution and invasive tendency, is not considered to be threatened at species level.

## Supplementary Material

XML Treatment for Venezillo parvus
